# Notes and News

**Published:** 1894-10-20

**Authors:** 


### Notes and News.

Collected and Communicated.

Professor Gussenbauer, of Prague, has been elected president of the German Surgical Congress for 1895.

Dr. Louis Vintras has been elected physician to the French Hospital, Dr. Armand Rüffer having resigned.

A new wing is to be added to the Swindon Victoria Hospital; the foundation stone was laid at the close of last month.

It is stated that the Local Government Board have decided not to hold an inquiry into the allegations made against the management of the Norwich Isolation Hospital.

A homœopathic hospital is about to be erected at Paris. This institution will owe its establishment to the will of Madame Henriette d'Argent, who bequeathed for this purpose a legacy of £2,000.

In consequence of the increased number of diphtheria cases seeking admission to the Fountain Hospital, two wards, containing forty-eight beds, have been prepared for the reception of diphtheria patients.

The system of pay wards at the Great Northern Hospital appears not to have found favour amongst some of the local medical men. It is stated that a protest will be made against the arrangement that paying patients shall be treated by the medical officers of the hospital free of charge, instead of extra payment being made either to these or to any private practitioner whom the patient may choose.

An interesting ceremony took place recently at Norwich, on the occasion of unveiling a memorial medallion terra-cotta bust of Jenny Lind at the hospital which bears her name, and which was founded through her generosity and energy. The Mayoress of Norwich unveiled the bust, which is a replica of that placed to the famous singer's memory in Westminster Abbey. The presence of Madam Albani added interest to the ceremony. The medallion is the gift of Mr. Otto Goldschmidt.

The Harveian Oration was delivered on Thursday, the 18th inst., at the Royal College of Surgeons, by Dr. T. Lauder Brunton, at four p.m.

The average cost per bed at the St. Petersburg Hospitals is £28 10s. The out-patients cost about one shilling per head.

The new Craig House, in connection with the Royal Edinburgh Asylum for the insane, is to be opened on the 26th inst. by the Duke and Duchess of Buccleuch. The present addition to the hospital consists of two wards, each containing twenty beds. Additional accommodation for the medical staff and servants has also been provided.

For some time past *Truth* has been investigating the affairs of the Jubilee Hospital. The statements, which are most discreditable to the secretarial management, so far remain unrefuted.

The new wing of the Croydon General Hospital was opened last week by the Archbishop of Canterbury. This is the second addition which has been made to the original building, and this institution is now one of the largest in the vicinity of London.

The anti-toxin treatment for diphtheria continues to spead. Two children's hospitals in Paris have now adopted it, and a Paris journal has started a subscription to enable it to be adopted at another institution in the city. The Pasteur method of injection against anthrax is also gaining ground. In Russia it is largely in use, and now a laboratory is to be established in St. Peterburg and other centres.

An inquiry is now being held at the St. John's Industrial School, Walthamstow, on behalf of the Home Office, respecting the allegations of improper punishment at the schools, and as to the general management of the institution. It will be remembered that the Rev. Lord Arthur Douglas reported on the cruelty of the punishments he witnessed during his time of office as temporary chaplain at the school.

With the object of recognising in a permanent form the valuable services rendered to the Queen's Hospital, Birmingham, by Alderman G. J. Johnson, as the honorary solicitor to the charity and as an active friend to the hospital for thirty-two years, the Board of the hospital are raising a subscription to present Alderman Johnson with a portrait to be placed in the board-room of the hospital, together with a replica of the same to be presented to Mrs. Johnson.

Where our anti-microbe measures are to end becomes a problem. A worthy F.R.C.S. sees in the postman's person a new danger. This possibly unhealthy mortal has handled our letters, therefore it is suggested that all letters should be dipped in a basin of disinfectant. But as postmen are only as liable to disease as other messengers, public and private, what an awful vista of danger opens before our enlightened eyes! The only safety seems to lie in a household quarantine scheme, followed by police observation for a given period.

